# 1720. Immunogenicity of COVID-19 Vaccines in Youth with Inflammatory Bowel Disease

**DOI:** 10.1093/ofid/ofad500.1552

**Published:** 2023-11-27

**Authors:** Blake T Cirks, Emily Parsons, Emily Samuels, Eric D Laing, Michael Rajnik, Allison M Malloy

**Affiliations:** Madigan Army Medical Center, Tacoma, Washington; Uniformed Services University, Bethesda, Maryland; Uniformed Services University, Bethesda, Maryland; Department of Microbiology and Immunology, Uniformed Services University, Bethesda, MD, Bethesda, Maryland; Uniformed Services University of the Health Sciences, Bethesda, MD; Department of Pediatrics, Uniformed Services University of the Health Sciences, Bethesda, MD, USA, Bethesda, Maryland

## Abstract

**Background:**

Youth with special healthcare needs or on immunosuppression are at increased risk for severe COVID-19, yet evaluation of the immunogenicity of SARS-CoV-2 vaccines in this population has been limited. A quarter of individuals with inflammatory bowel disease (IBD) are diagnosed in childhood and require chronic immunosuppressive therapy. Describing the quality and duration of the humoral and cellular responses to novel mRNA vaccines in this population can improve implementation and protection for this vulnerable population.

**Methods:**

This ongoing single-center, prospective, longitudinal cohort study enrolled children aged 6–21 years at-risk for severe COVID-19 beginning in January 2023. On enrollment and at pre-specified times after vaccination peripheral blood samples, epidemiologic and clinical data were obtained. Total SARS-CoV-2 spike (S) and receptor binding domain (RBD)-specific IgG antibodies, as well as endemic coronavirus (eCoV) antibodies, were measured using a multiplex microsphere immunoassay. SARS-CoV-2 specific T cell responses were measured with intracellular cytokine staining (ICS) after peptide stimulation.

**Results:**

11 SARS-CoV-2-immunized children with IBD treated with anti-tumor necrosis factor (TNFα) therapy were enrolled (Table 1). The seroconversion rate was 100% (Figure 1). Anti-S IgG levels increased with more exposures (r =.81 p =.003) and decreased over time with a negative correlation between anti-S IgG and days since last exposure (r = -.691 p =.02), as well as a significant difference in anti-S IgG when grouped by time since last exposure (p =.02) (Figure 2). ECoV antibody levels did not significantly correlate with anti-S IgG. 63% and 81% of participants developed SARS-CoV-2 specific CD4+ and CD8+ T cell responses, respectively, though response magnitude was low (Figure 1). A majority of participants demonstrated measurable antibody (100%) and T cell (90%) responses > 12 months after their last vaccination.

Table 1

**Figure:**
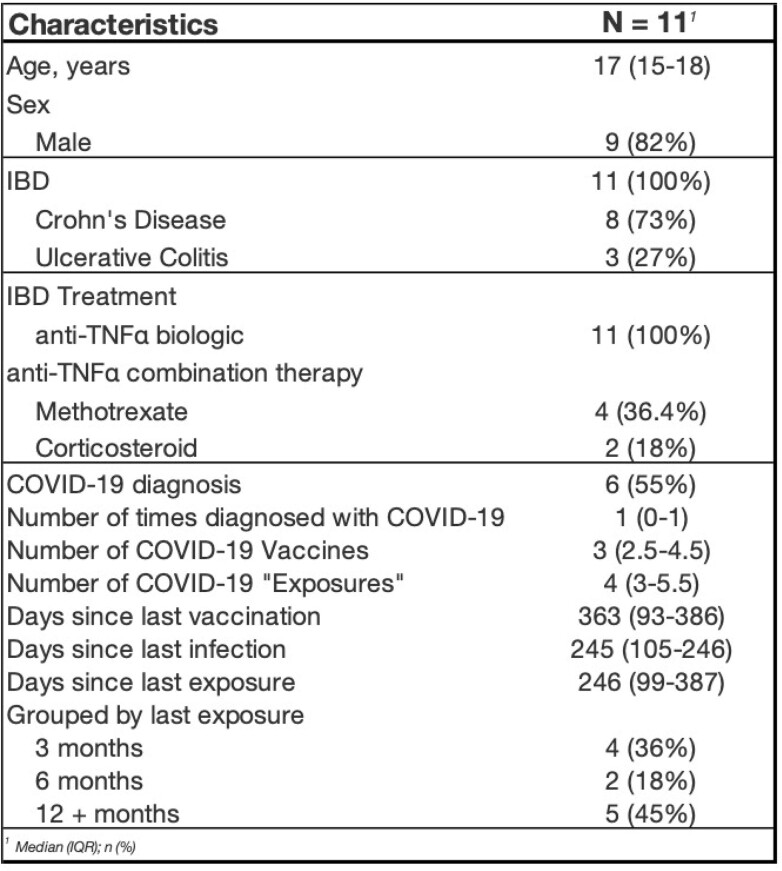

Demographics of Enrolled Research Subjects

Figure 1

**Figure:**
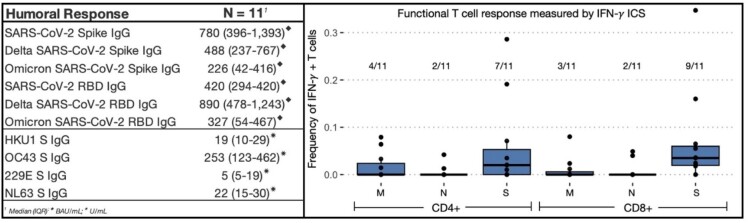

Cohort Humoral and Cellular Responses. Antibody levels (left) of SARS-CoV-2 wild type plus Delta and Omicron variants in BAU/mL. ECoV antibody levels in U/mL. T cell response (right) evaluated by frequency of CD4+ and CD8+ IFN-γ+ T cells following stimulation with wild type SARS-CoV-2 S, N, M peptides.

Figure 2

**Figure:**
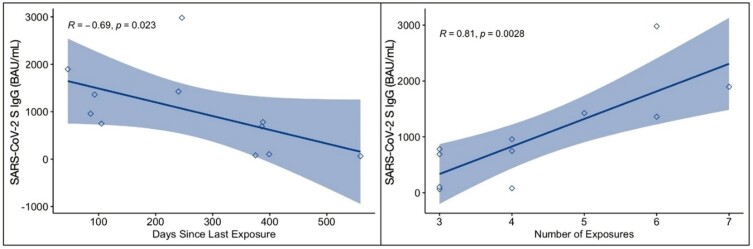

Humoral Immune Durability and Response. Significant correlation between SARS-CoV-2 S IgG and days since last exposure (left) and SARS-CoV-2 S IgG and number of exposures (right). Exposure = vaccination or infection. Shaded region 95% CI, n=11.

**Conclusion:**

Our small, ongoing cohort study demonstrates both SARS-CoV-2 antibody and T cell responses can develop following vaccination in children with IBD on anti-TNFα therapy. While the humoral response decreased over time, a durable cellular response persisted at 12 months in most participants.

**Disclosures:**

**All Authors**: No reported disclosures

